# The Values and Limitations of FDG-PET/CT for Diagnosis of Hibernoma

**DOI:** 10.1155/2015/958690

**Published:** 2015-12-13

**Authors:** Jong Hoon Park, Koichi Ogura, Tomohiro Fujiwara, Akihito Nagano, Kunihiko Numoto, Takashi Terauchi, Akihiko Yoshida, Akira Kawai

**Affiliations:** ^1^Department of Musculoskeletal Oncology, National Cancer Center Hospital, Tokyo 104-0045, Japan; ^2^Department of Orthopaedic Surgery, Korea University Hospital, Seoul 02841, Republic of Korea; ^3^Department of Nuclear Medicine, National Cancer Center Hospital, Tokyo 104-0045, Japan; ^4^Department of Pathology, National Cancer Center Hospital, Tokyo 104-0045, Japan

## Abstract

Hibernoma is a rare benign lipogenic tumor of brown fat that develops in a wide variety of locations. Although the features of hibernoma demonstrated by MRI resemble those of liposarcoma, recent FDG-PET/CT studies have documented higher radiotracer uptake than liposarcoma, suggesting that FDG/PET/CT is useful for differentiating hibernoma from liposarcoma. Here we report two cases of hibernoma that showed relatively lower SUVs than those reported previously, lying within the range for liposarcoma. Our findings emphasize that hibernoma needs to be included in the differential diagnosis of any fat-containing tumor showing intense accumulation by FDG-PET/CT. Although it is unlikely that such a rare condition could be reasonably diagnosed on the basis of MRI and FDG-PET/CT alone due to possible SUV overlap between hibernoma and liposarcoma, it is important to recognize this extremely rare lipogenic tumor for accurate diagnosis and appropriate management.

## 1. Introduction

Hibernoma is a rare benign tumor of brown fat that usually occurs in adults with a slight male preponderance [1]. It develops in a wide variety of locations, including the thigh, shoulder, axilla, mediastinum, and upper thorax. It is a benign tumor that does not recur after complete excision [1].

[^18^F]2-Deoxy-2-fluoro-D-glucose (FDG) positron emission tomography (PET) has been proven to be a valuable imaging method for evaluating the functional characteristics of soft tissue masses and providing information that is critical for patient management. In a FDG-PET study of 120 soft tissue tumors, Aoki et al. found a statistically significant difference (*P* < 0.0001) of standardized uptake value (SUV) between benign and malignant soft tissue tumors [2]. Recently, despite its benign nature, hibernoma has been recognized to demonstrate an extremely high SUV in FDG-PET/CT due to high metabolic activity [3–5]. These previous reports concluded that awareness of these radiographic features might allow differentiation of hibernoma from liposarcoma, since hibernoma shows a much higher SUV than liposarcoma [6–8]. However, this hypothesis is based on only a limited number of cases, and the diagnostic value of FDG-PET/CT for hibernoma is not yet well defined. Herein we present two cases of hibernoma that showed relatively lower SUVs than those reported previously, lying within the range for liposarcoma, and based on these cases we sought to define the utility of FDG-PET/CT for diagnosis of hibernoma with reference to the available literature.

## 2. Case Report

### 2.1. Case  1

**Figure 1:**
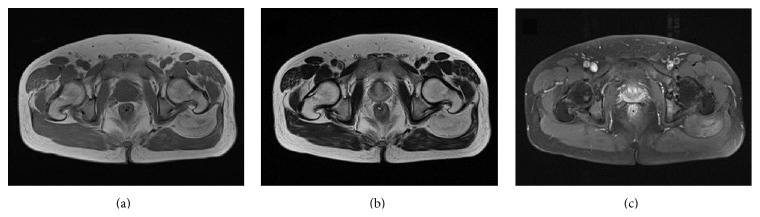
Axial MRI showing a well-circumscribed slightly heterogeneous mass between the gluteus medius and maximus muscles. On both T1-weighted (a) and T2-weighted (b) images, the signal intensity of the tumor appears high relative to skeletal muscle, being suggestive of fat. Diffuse contrast enhancement is evident (c).

**Figure 2:**
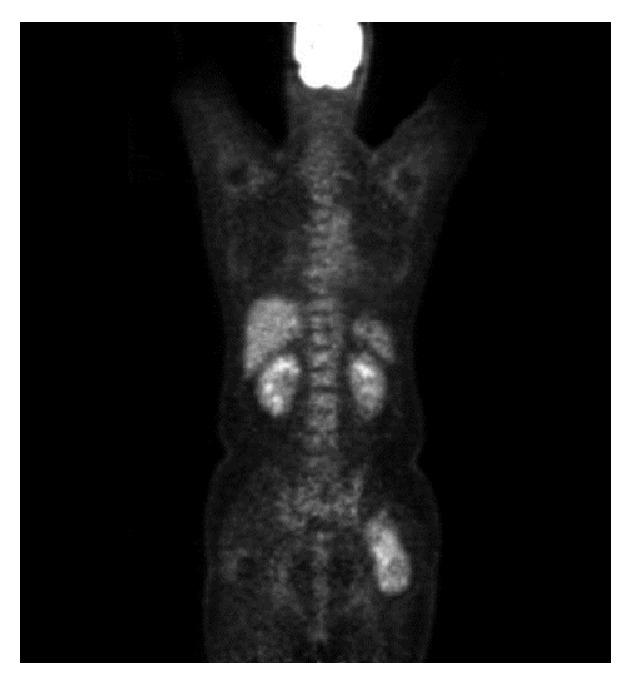
Coronal FDG-PET/CT demonstrating intense FDG uptake (SUVmax 4.1) within the tumor.

A 42-year-old man presented at our institution for evaluation of a left buttock mass that had been found incidentally at a general check-up. On physical examination, a nontender, deep-seated elastic soft tissue mass was palpable in his left buttock. There was no local lymphadenopathy. Tinel's sign was not present, and the patient had no discomfort attributable to the mass. Magnetic resonance imaging (MRI) revealed a well-circumscribed slightly heterogeneous mass 12 cm in diameter between the gluteus medius and maximus muscles. On both T1- and T2-weighted images, the signal intensity of the tumor was considerably higher than that of skeletal muscle but slightly lower than that of subcutaneous fat (Figures 1(a) and 1(b)). On fat-suppressed T1-weighted images, the mass showed a mixture of low and intermediate signals, compatible with signal suppression from the fatty elements of the tumor. Diffuse contrast enhancement was observed after intravenous administration of contrast medium (gadolinium-diethylenetriamine penta-acetic acid (Gd-DTPA)) (Figure 1(c)). FDG-PET/CT showed increased FDG uptake (SUVmax 4.1) (Figure 2). No other abnormal FDG uptake was observed.

**Figure 3:**
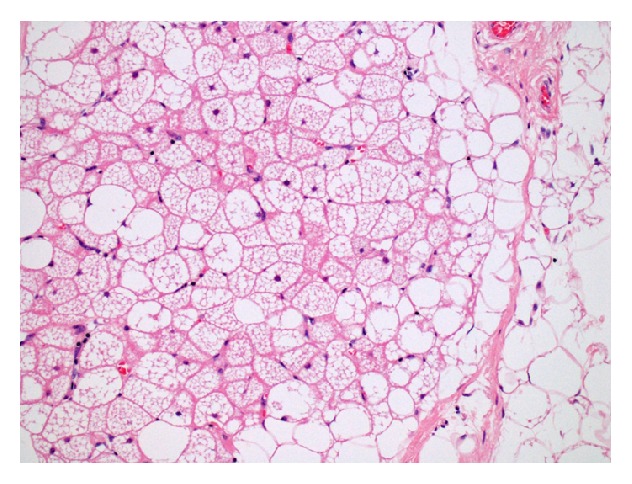
The tumor is composed of a mixture of brown fat (hibernoma) cells and mature white fat cells (H&E, ×200).

Core needle biopsy was performed and a histological diagnosis of hibernoma was made. The patient underwent excision of the tumor without any complications. Macroscopically, the tumor was yellow tan in color and had a fatty consistency. Microscopically, a mixture of brown fat cells (hibernoma cells) and mature white fat cells was observed. No cellular atypia or mitotic figures were observed. As the hibernoma cells were scattered among white fat cells, the tumor was classified as the lipoma-like variant (Figure 3). After 8 months, no local recurrence of the tumor had been detected.

### 2.2. Case  2

**Figure 4:**
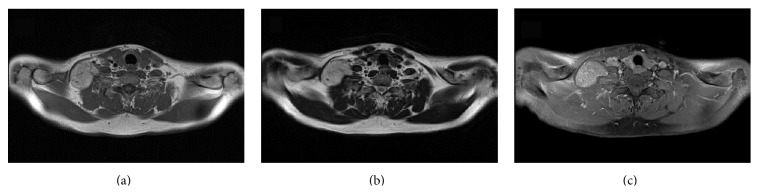
Axial MRI showing an irregularly shaped mass in the supraclavicular region. On both T1-weighted (a) and T2-weighted (b) images, the signal intensity of the tumor appears high. The tumor shows moderate enhancement throughout (c).

**Figure 5:**
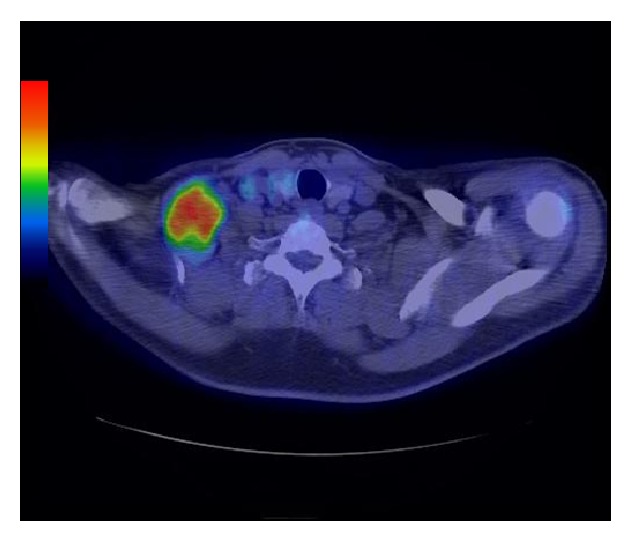
Axial FDG-PET demonstrating intense FDG uptake (SUVmax 6.4).

A 50-year-old man presented at our institution for evaluation of a painless soft tissue mass in the anterior neck that had been present for three years. On physical examination, a nontender, egg-sized elastic soft mass was palpable in the right supraclavicular region. There was no local lymphadenopathy, and Tinel's sign was not present. Magnetic resonance imaging revealed a well-circumscribed mass 7 cm in diameter with a slightly irregular shape in the supraclavicular region. On both T1- and T2-weighted images, the signal intensity of the tumor was significantly higher than that of skeletal muscle but slightly lower than that of subcutaneous fat (Figures 4(a) and 4(b)). Fat-suppressed T1-weighted images obtained after administration of gadolinium contrast material showed moderate enhancement throughout the tumor and linear and curvilinear structures with increased signal intensity corresponding to large intratumoral vessels (Figure 4(c)). Contrast-enhanced CT scans showed a predominantly low-attenuation mass with curvilinear branching blood vessels. FDG-PET/CT demonstrated increased FDG uptake (SUVmax 6.4) (Figure 5). No other abnormal FDG uptake was observed. Core needle biopsy was performed and a histological diagnosis of hibernoma was made. Conservative observation with serial MRI and CT demonstrated no significant change in the size or appearance of the tumor for 2 years.

## 3. Discussion

Hibernoma is a rare benign soft tissue tumor originating from brown fat. It usually manifests as a slowly growing and painless soft tissue mass with a peak incidence in the fourth and fifth decades of life. There is a slight male preponderance [1]. The name “hibernoma” was derived from its morphological resemblance to the brown fat in hibernating animals. Brown fat is a specialized form of adipose tissue that acts to generate heat in response to cold exposure (nonshivering thermogenesis) and ingestion of food (diet thermogenesis). The volume of brown fat normally diminishes shortly after birth, but it may persist in some locations such as the neck, axilla, paraspinal area, intercostal spaces, and retroperitoneum. Hibernoma can occur in any location where brown fat remains [1]. Histologically, hibernoma is composed of multivacuolated adipocytes with small central nuclei and an increased number of mitochondria, mixed with variable proportions of univacuolated adipocytes resembling normal adult adipose tissue.

On CT and MRI, hibernoma usually manifests as a heterogeneous fat-containing mass with internal septations or fine enhancing strands. A large branching vessel is frequently found within the lesion after intravenous administration of contrast medium [9]. Although the likelihood of confusing hibernoma with other tumors is minimal on the basis of pathologic examination, it often shows radiological features that can be confused with those of malignant lesions. Radiologically, the differential diagnosis of hibernoma would include several fat-containing lesions such as liposarcoma and lipoma and subtypes of lipoma such as angiolipoma, since the abovementioned imaging characteristics are not completely specific. Therefore, it has been considered difficult to differentiate hibernoma from liposarcoma on the basis of imaging findings alone, and pathologic evaluation is required for confirmation [10–13].

Recently, several reports have suggested that the SUV shown by FDG-PET/CT can be used to differentiate hibernoma from liposarcoma [3–8, 14]. Previous studies of FDG uptake in lipogenic tumors have shown that liposarcoma lies in the low to intermediate range (0.37–9.1), whereas lipoma shows low uptake (<2.0) [2, 15]. Since hibernoma has been reported to show a much higher SUV (>10) than liposarcoma, it has been suggested that SUV would be potentially useful for differentiating these entities [3–8, 14]. The intense FDG uptake shown by hibernoma despite its benign nature is considered to be attributable to the high rate of glucose metabolism in brown adipose cells within the tumor rather than to tumor growth activity itself.

Although the SUVs of the two hibernomas we investigated were certainly high, as reported previously [3–8, 14], they were relatively lower than those reported in previous cases, lying within the range for liposarcoma [15]. One possible reason for the difference between our present findings and those of previous studies may lie in the small number of cases (only 11) examined so far, the cases showing high SUVs perhaps being merely incidental. Another possible explanation is that SUVs may fluctuate over time, as suggested by Smith et al. [16], and that hibernoma may demonstrate a wide SUV range. According to Cohade et al. [17], one of the characteristics of brown fat is its variability of FDG uptake over time; it may be momentarily intense, but 2 weeks later it may be absent. The most acceptable explanation for this phenomenon is that brown fat is responsive to ambient temperature, its FDG uptake increasing during cooler periods of the year. Another study has shown that controlling a patient's environmental temperature before injection of a radiotracer and during the uptake phase can markedly change FDG uptake in areas of brown fat [18].

From our experience of the present two cases and previous series, it is evident that hibernoma shows a wide variation of SUV in the high range relative to lipoma. However, it is difficult to distinguish hibernomas from liposarcomas on the basis of FDG-PET/CT findings alone, as the SUVs of hibernoma can overlap with those reported for liposarcoma. In view of the limited data currently available, further studies including a large number of cases are needed to clarify the clinical value of FDG-PET/CT for diagnosis of hibernoma.

In summary, our experience with the present two cases emphasizes the need to include hibernoma in the differential diagnosis of any fat-containing tumor showing intense accumulation in FDG-PET/CT examination, as the SUV reflects metabolic activity and cellular components, rather than malignant potential. Although it is unlikely that this rare lipogenic tumor could be reasonably diagnosed on the basis of MRI and FDG-PET/CT findings alone due to the considerable overlap of SUV between hibernoma and liposarcoma, it is important for clinicians to be aware of it in order to arrive at an accurate diagnosis, provide appropriate management, and avoid any unnecessary cytotoxic or surgical treatment.
